# Modulation of physicochemical properties of lipid droplets using soy protein isolate and lactoferrin interfacial coatings

**DOI:** 10.1002/fsn3.3723

**Published:** 2023-10-17

**Authors:** Chunlan Zhang, Mengyao Du, Bin Li

**Affiliations:** ^1^ College of Food Science and Engineering Tarim University Alar China; ^2^ Production and Construction Group Key Laboratory of Special Agricultural Products Further Processing in Southern Xinjiang Alar, Xinjiang China; ^3^ College of Food Science and Technology Huazhong Agricultural University Wuhan China

**Keywords:** electrostatic interaction, in vitro digestion, multilayer emulsion, physicochemical property, stability

## Abstract

In order to improve the physicochemical stability of soy protein isolate (SPI) emulsion, lactoferrin (LF) was used to modify the interface layer. The stable multilayer emulsion can be formed when the content of lactoferrin is 0.5% at pH 5. The emulsion with good stability was at pH 3–7, and it was also stable to temperature change. The FFAs release of SPI emulsion and LF‐SPI emulsion was 103.9% and 103.7%, respectively. The results showed that the lactoferrin layer did not hinder the digestion of oil and the bioaccessibility of carotenoids, but lactoferrin layer improved the physicochemical stability of SPI emulsions. This work provides information valuable in the design of emulsion formulations for applications in the food, pharmaceutical, and personal care industries.

## Graphical abstract

1. Emulsion‐based delivery systems were prepared consisting of lipid droplets covered by soy protein isolate (SPI) and lactoferrin (LF) coatings.
2. The emulsion with good stability was at pH 3–7, and it was also stable to temperature change.
3. The lactoferrin layer did not hinder the digestion of oil and the bioaccessibility of carotenoids.

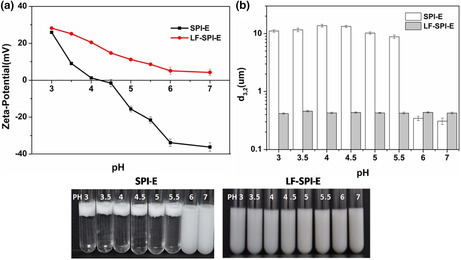

## 1 INTRODUCTION

Carotenoids are pigments derived from plants and microorganisms that play a very important role in biological systems. They have many health benefits that attract the interest of researchers and consumers (Roll Zimmer et al., 2022). However, they are chemically unstable; stability is affected by light, acid, oxygen, and heat and displays insufficient water solubility, leading to poor processing adaptability, which restricts their application in food and pharmaceutical products (Li et al., 2022; Liu & Liu, 2022). To overcome the limitations, a useful strategy is used to encapsulate carotenoids into appropriate delivery vehicles (Geng et al., 2022). Using β‐lactoglobulin and Tween‐20 as emulsifiers and orange oil as oil phase, Cheng et al. prepared beta‐carotene nanoemulsions with small particle size and good stability using high‐pressure homogenization; the beta‐carotene degradation rate in β‐lactoglobulin nanoemulsion is slower than that in Tween 20 nanoemulsion (Qian et al., 2012a). Rao et al. used sucrose monoester and lecithin as mixed emulsifiers, and corn oil and lemon oil as the oil phase of lipid digestion to prepare beta‐carotene emulsion by high‐pressure homogenization (Rao et al., 2013). Lv et al. studied lycopene oil solution (0.8 wt%) as oil phase, whey protein isolate (WPI) solution (0.6 wt%) as first layer, and chitosan solution (0–1.50 wt%) as second layer; lycopene‐loaded double‐layer emulsion was prepared. The result proved that the bilayer emulsion could provide better protection for lycopene encapsulated than the WPI monolayer emulsion (Lv et al., 2021).

Double‐layer emulsion is a kind of multilayer emulsion, which is formed by layer‐by‐layer electrostatic self‐assembly technology; the two interface layers can be formed on the surface of emulsion droplets by electrostatic adsorption or covalently binding of biopolymers such as protein, polysaccharide, or small molecular surfactant such as phospholipids and Tween (Liu et al., 2022). As a transfer system, double‐layer emulsion has more advantages in structural stability and resistance to environmental pressures (such as pH, temperature, and ionic strength) than traditional single‐layer emulsion, the invention can provide better protection for the embedded bioactive substances, and has certain controlled release ability (Shen et al., 2023). The research shows that two different proteins can be used to prepare double‐layer emulsions. Researchers have tried using lactoferrin and β‐lactoglobulin with isoelectric points of 8.5 and 5.0, respectively, to make a double‐layer emulsion (Schmelz et al., 2011). Mao et al. found that the double‐layer emulsion prepared by lactoferrin and β‐lg had better thermal stability at 90°C than the emulsion prepared by using the mixture of the two proteins as emulsifier. The double‐layer emulsion has a good protective effect on sensitive lipophilic active components (Mao et al., 2013). Lesmes et al. prepared bilayer emulsions with sodium casein, and lactoferrin‐embedded omega‐3 fatty acid has good physical stability and the oxidation stability of the bilayer emulsions is significantly improved compared with that of the primary emulsions prepared with sodium casein only (Lesmes et al., 2010).

Soy protein isolate contains more than 90% protein, nearly 20 kinds of amino acids and human essential amino acids. Its nutrient‐rich, cholesterol‐free, soy protein isolate is one of the few alternatives to animal protein varieties (Shen et al., 2022; Zhang et al., 2022). Because of its good surface activity, it can reduce the surface tension of water and oil and reduce the surface tension of water and air, and it easily forms a stable emulsion; therefore, soy protein isolate is widely used in food (Du et al., 2022; Ma et al., 2022). However, because the isoelectric point of the protein is about pH 4.5, the emulsion prepared with soy protein isolate is unstable and prone to droplet aggregation under acidic conditions (Wang, Wang, et al., 2022; Zhang & Li, 2021). Therefore, in order to improve the stability of SPI emulsion, lactoferrin was used to modify the interface layer. Lactoferrin is an iron‐binding glycoprotein, which contains about 680 amino acid residues with a molecular weight of about 80 kDa. The isoelectric point of lactoferrin was 8.5, so the protein was positively charged in the neutral solution. Because of its good surface activity, lactoferrin is used as a model globulin assembly interface layer. At the same time, lactoferrin has potential health benefits, such as anticancer, antioxidant, anti‐inflammatory, and antimicrobial activities (Wang, Li, et al., 2022; Wei et al., 2022; Xia et al., 2022).

Therefore, in this study, using carotenoid as bioactive substance, which was extracted from *Lycium barbarum*, the primary emulsion was prepared with soy protein isolate, and lactoferrin was used to modify the interface layer of SPI emulsion droplets for improving the physicochemical stability in order to provide a theoretical basis for the practical application of the emulsion system.

## 2 MATERIALS AND METHODS

### 2.1 Materials

Soy protein isolate (protein content was reported to be 90%, dried weight) was obtained from Yuanye Bio‐Technology Co. Ltd. Food‐grade lactoferrin (contained 95% protein and 15% iron saturation) was supplied by DMV International. Twenty percent of carotenoids from *L. barbarum* was prepared by extracting with hexane solvent. Medium‐chain triglyceride (MCT) was obtained from Boxing Chemical Reagent Co. Ltd. Sodium azide, ethanol, and *n*‐hexane were purchased from Sinopharm Chemical Reagent Co. Ltd. All other chemicals were of analytical grade unless otherwise stated. Deionized water obtained from a Milli‐Q water purification system (Millipore Co.) was used in all experiments.

### 2.2 Emulsion preparation

#### 2.2.1 Preparation of primary emulsion

SPI solutions (1 wt%) were made by dissolving protein powders in 10 mM phosphate buffer (pH 7) at room temperature for 2 h with continuous stirring. A small amount of insoluble matter in the protein solutions was removed by centrifugating at 4000 rpm for 5 min. The primary emulsion was prepared by mixing 5 wt% MCT with 95 wt% SPI solutions using a high‐speed mixer (PT‐MR2100, Kinematica Co.) working at 26,000 rpm for 2 min. Next, the coarse emulsion was conducted by a high‐pressure homogenizer (M‐110L, Microfluidics) for five cycles at a pressure of 75 Mpa. The homogenizer was chilled throughout the homogenization procedure to avoid excessive heating of the emulsions. Primary emulsions were then diluted on a 1:4 mass ratio with 10 mM phosphate buffer solution (pH 7.0).

#### 2.2.2 Preparation of multilayer emulsion

LF solutions (2 wt%) were made by dissolving protein powders in 10 mM phosphate buffer (pH 7) at room temperature for 2 h with continuous stirring. The diluted primary emulsion containing 1 wt% MCT and 0.2 wt% SPI was mixed with same volume lactoferrin solutions and the final concentration of lactoferrin was 0%–0.9%. Then, the emulsions were adjusted from pH 7.0 to 5.0 by the addition of HCl or NaOH to promote the adsorption of the lactoferrin onto the oppositely charged droplet surfaces. The emulsions were stirred for 30 min and the resulting emulsions containing SPI and lactoferrin were referred to as multilayer emulsions. Then, the multilayer emulsion was stored at ambient temperature for 24 h prior to analysis.

### 2.3 Exposure to environmental stresses

The influence of environmental stress on the stability of primary and multilayer emulsions was tested. The pH stability was determined by adjusting the aqueous phase of the emulsions to values ranging from 3.0 to 7.0 by adding NaOH or HCl, respectively. The salt stability was determined by adding 0–500 mM NaCl to the primary emulsions at pH 7.0 and multilayer emulsions at pH 5.0. The thermal stability was determined by taking 10 mL samples in a water bath heated to fixed temperatures ranging from 50 to 90°C for 30 min, and then cooling them to ambient temperature. The treated samples were stored for 24 h at ambient temperature before being analyzed.

### 2.4 Carotenoid content determination

Samples were stored at 37°C to accelerate carotenoid degradation. The carotenoid content in the emulsion was measured during the storage following the method (Yuan et al., 2008). The absorbance was measured at 450 nm using a spectrophotometer (UV‐1100, Meipuda Instrument). Total carotenoid content was calculated according to McBeth's formula as (Lin et al., 2011):

(1) $$\text{Carotenoids}\;\left(\mathrm{mg}/100\;g\right)=\frac{A\times V\times 1000}{E_{1\;\mathrm{cm}}^{1\%}\times m}$$

where absorbance (*A*) was determined by spectrophotometer at 450 nm, *V* was the total volume of the solution (mL), $E_{1\;\mathrm{cm}}^{1\%}$ was 1% of the average extinction coefficient value of carotenoids in *n*‐hexane (2500), and m was the weight of the emulsion (g).

The carotenoid content was expressed as retention rate relative in percent: *C*(*t*)/*C*_0_ (%), where *C*(*t*) is the carotenoid concentration after storage for a period *t* and *C*_0_ is the initial carotenoid concentration.

### 2.5 Droplet size and surface charge measurements

The droplet size was measured by the static light‐scattering instrument (Mastersize2000, Malvern Instruments). The droplet size measurements are reported as the surface‐weighted mean diameter: *d*_3,2_ = ∑ni3/∑ni2, where ni is the number of droplets of diameter di. The ζ‐potential of the emulsions was measured using a particle mobility distribution instrument (Zetasizer Nano ZS Malvern Instruments). To avoid multiple scattering effects, emulsions were first diluted 100 times using buffer solutions of the appropriate pH and salt concentration.

### 2.6 In vitro digestion

To determine the free fatty acids (FFAs) released in emulsions, a dynamic in vitro digestion procedure with simulated gastrointestinal tract was conducted with a classic method (Sarkar, Goh, & Singh, 2009; Sarkar, Goh, Singh, & Singh, 2009). The automatic titration experiment was started and the pH was maintained by pH‐stat (907 Titrando, Metrohm, USA Inc.). The amount of FFAs released during the digestion process was calculated by the following equation (Salvia‐Trujillo et al., 2013).

(2) $$\text{FFAs}\;\text{Released}\;\left(\%\right)=\frac{C_{\text{NaOH}}\times V_{\text{NaOH}}\times M_{\text{oil}}}{2\times m_{\text{oil}}}\times 100$$

where *C*_NaOH_ is the molarity of the NaOH solution used to titrate the sample (mol/L), *V*_NaOH_ is the volume of NaOH solution required to neutralize the FFAs produced at digestion, *m*_oil_ is the total mass of oil initially present in the incubation cell (g), and *M*_oil_ is the molecular weight of the oil (the molecular weights of the MCT were taken to be 500 g/mol).

### 2.7 Bioaccessibility determination

The bioaccessibility of carotenoids in the nanoemulsions was determined after the in vitro digestion process using a method described previously (Qian et al., 2012b). An aliquot of the raw digesta was centrifuged at 16,000 rpm for 30 min at 4°C. The middle transparent layer was taken to be the “micelle fraction” in which the carotenoids were solubilized. The concentrations of carotenoids were measured by 2.4. The bioaccessibility was calculated using the following equation:

(3) $$\text{Bioaccessibility}\left(\%\right)=\frac{C_{\text{micelle}}}{C_{\text{digesta}}}\times 100$$

where *C*_micelle_ and *C*_digesta_ are the concentrations of carotenoids in the micelle fraction and in the overall sample (raw digesta) after the pH‐stat experiment, respectively.

### 2.8 Statistical analysis

All experiments were carried out in triplicate using freshly prepared samples. All results are presented as the calculated mean and standard deviations.

## 3 RESULTS AND ANALYSIS

### 3.1 Influence of lactoferrin concentration on the primary emulsion

**FIGURE 1:**
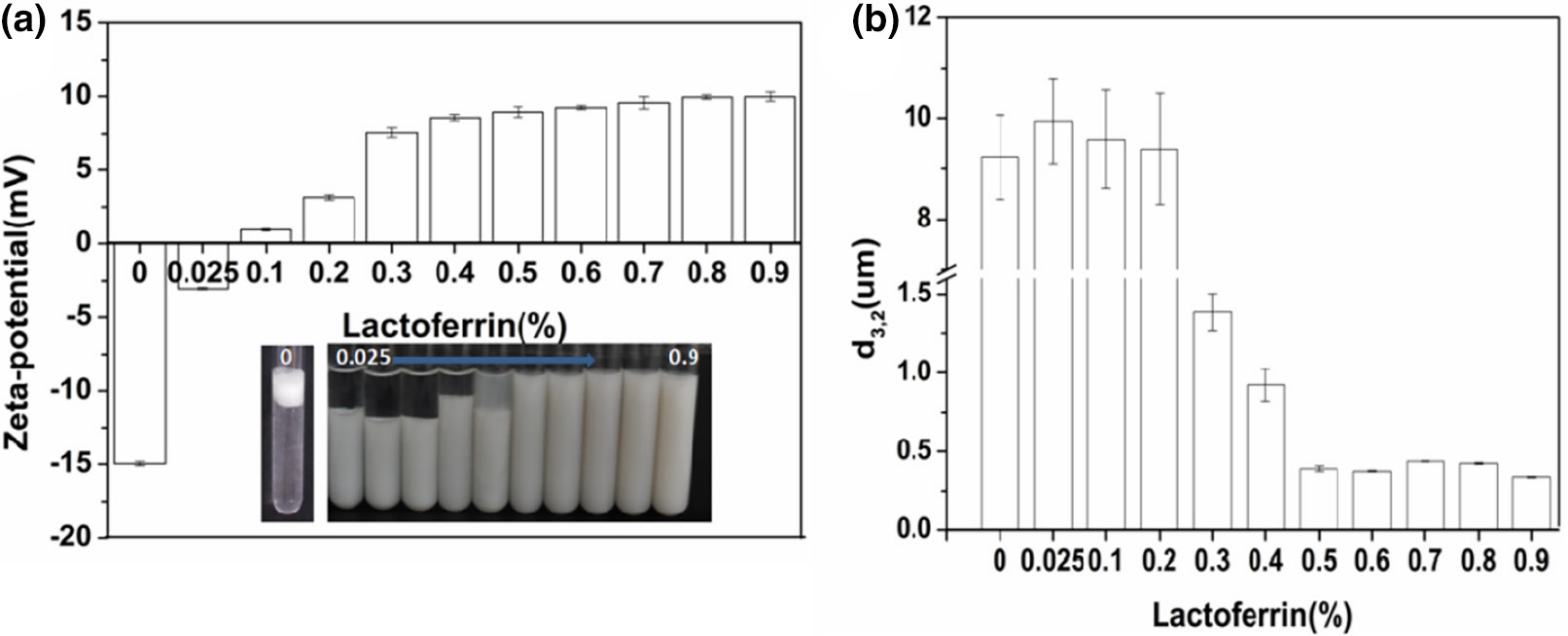
Effect of lactoferrin concentration on the zeta‐potential (a) and droplet size (b) of SPI‐stabilized emulsion at pH 5. SPI, soy protein isolate.

Because isoelectric point of lactoferrin was 8.5 and isoelectric point of SPI was 4.5 (Ye & Singh, 2007), these two proteins tend to form electrostatic complexes over a fairly large pH range, so pH 5 was chosen to prepare multilayer emulsions. The primary emulsion has a negative charge (−15.7 ± 0.1 mV) at pH 5. With the increase in lactoferrin content, the emulsion charge gradually increases to a positive charge, and then it flattens out (≈+10 mV). This was mainly due to the negative charge on the surface of the primary emulsion being neutralized by the positive charge of lactoferrin. Lactoferrin was adsorbed on the surface of the primary emulsion through electrostatic interaction. The potential value was basically stable when lactoferrin content ≥0.5% (Figure 1a). It indicated that lactoferrin has fully saturated the surface of primary emulsion. Under low concentrations of lactoferrin, bridging flocculation was easy to occur. When the content of lactoferrin was ≥0.5%, the emulsion was relatively stable without flocculation, and the droplet size decreased (*d*_3,2_ ≈ 0.4 μm). The stable multilayer emulsion can be formed when the content of lactoferrin is 0.5%.

### 3.2 Influence of protein coatings on pH stability

**FIGURE 2:**
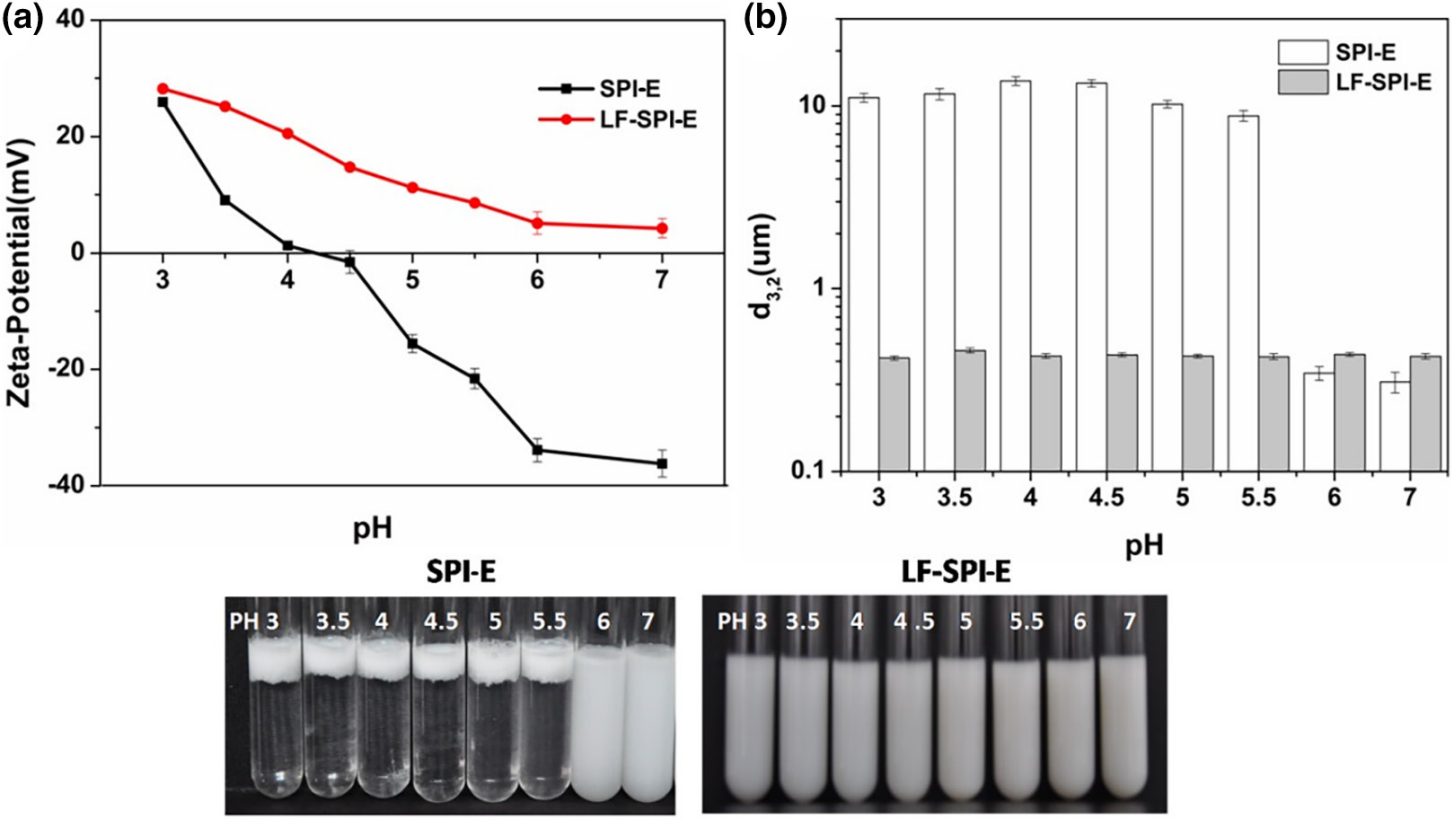
Effect of pH on the zeta‐potential (a) and droplet size (b) of emulsions. SPI, soy protein isolate.

Emulsion‐based delivery systems may be incorporated into food and beverage products that have different pH values. Therefore, the influence of pH on the electrical characteristics and stability of emulsions were examined. The two emulsions exhibited appreciable differences in their stability to pH changes (Figure 2). The surface charge in the SPI emulsions changed from highly positive to highly negative (+25.9 to −39.2 mV) as the pH was increased from 3 to 7, with a point of zero charge around pH 4.5 (Figure 2a), which was consistent with the previous reports (Jaramillo et al., 2011). There was a substantial charge decrease in the multilayer emulsion with increasing pH from 3 to 7 (+28.2 to +4.24 mV), which was attributed to the isoelectric point of LF is 8.5 (Levay & Viljoen, 1995).

Primary emulsions containing SPI‐coated lipid droplets were relatively stable to droplet aggregation at pH values (pH 6 and 7) sufficiently above the adsorbed protein's isoelectric point (pI ≈ 4.5), which can be attributed to strong electrostatic repulsion between SPI‐coated droplets in these pH ranges. However, they were highly unstable in acid conditions. As reported previously, this effect can be attributed to the fact that the van der Waals attraction was sufficiently strong to overcome the weak electrostatic repulsion between the droplets, thereby leading to aggregation (Demetriades et al., 1997a). In contrast, multilayer emulsions were relatively stable to aggregation across the entire pH range studied (pH 3–7) (Figure 2b). The electrostatic repulsion can maintain the emulsion stability at acid conditions. However, the multilayer emulsions were also stable to aggregation even under conditions where the droplets had a relatively low net charge, that is, pH 6 and 7 (Figure 2a). Previous studies showed that steric repulsion plays an important role in determining the aggregation stability of LF‐coated droplets (Ye & Singh, 2007). On the one hand, LF molecules have a relatively high molecular weight (80 kDa) (Levay & Viljoen, 1995), so the steric repulsion between droplets coated by LF was longer range. On the other hand, LF is a surface‐active globular glycoprotein that has hydrophilic carbohydrate groups attached to the polypeptide chain and the containing hydrophilic carbohydrate side chains will protrude into the aqueous phase thereby increasing the strength of steric repulsion (Ye & Singh, 2007).

### 3.3 Influence of protein coatings on salt stability

**FIGURE 3:**
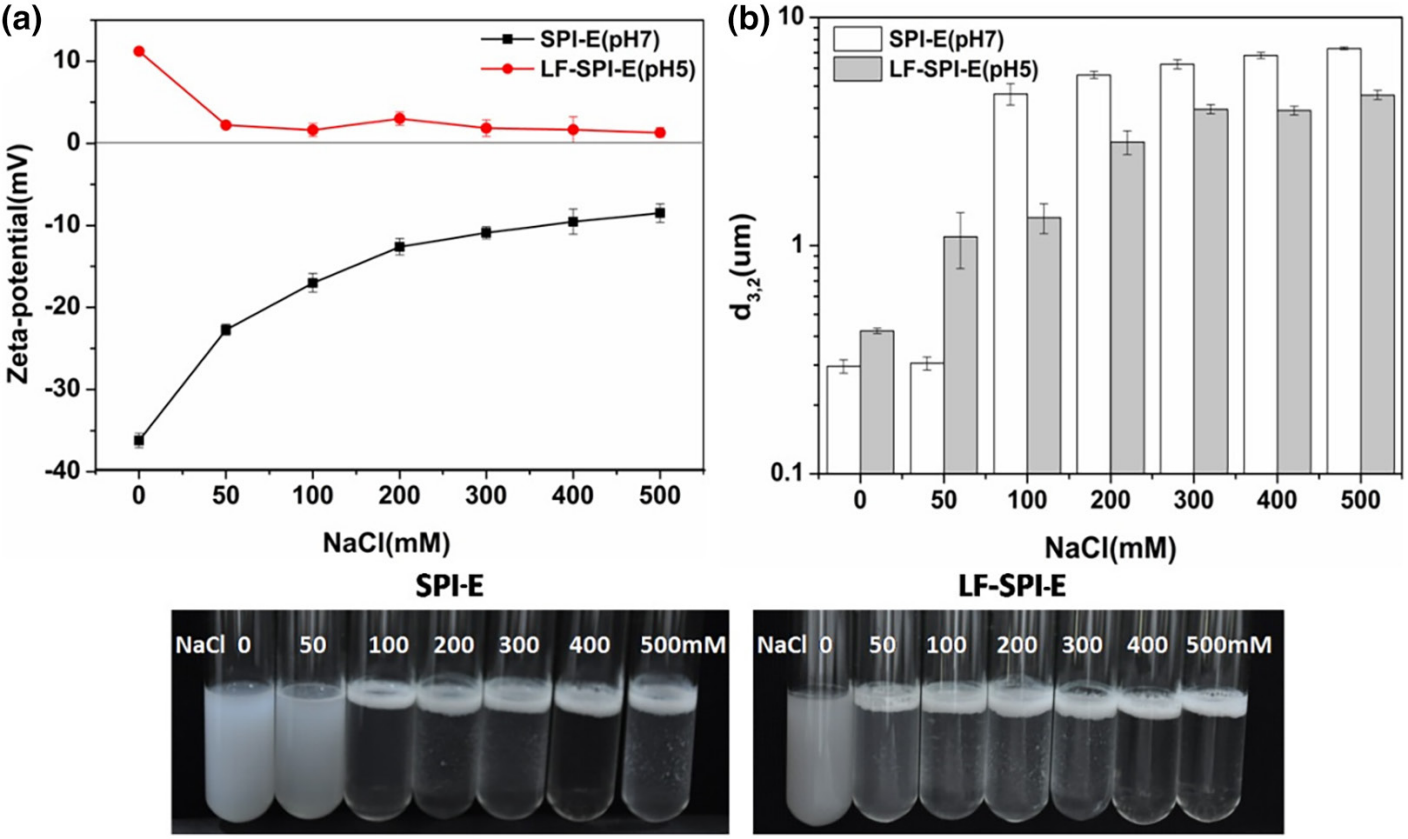
Effect of salt concentration on the zeta‐potential (a) and droplet size (b) of emulsions. SPI, soy protein isolate.

Salts, such as sodium chloride, were added to food products to improve taste, preservation, or modification of physicochemical properties. The influence of salt addition on the electrical characteristics and aggregation stability of the emulsion was measured. The magnitude of the electrical charge on all of the emulsions decreased with increasing salt concentration (Figure 3a), which can be mainly attributed to electrostatic screening effects of salt. The primary emulsions were unstable to salt addition, and there was droplet aggregation at high ionic strengths (≥100 mM NaCl). The droplet size of multilayer emulsion also increased with increasing NaCl concentration. This effect can be attributed to the ability of the counter‐ions in NaCl to screen the electrostatic repulsive forces acting between the lipid droplets (Lv et al., 2021). At relatively low ionic strengths, the electrostatic and steric repulsion are sufficiently strong. At higher ionic strengths, the electrostatic force was weakened, and the steric repulsion alone was not sufficiently strong to prevent the droplets from coming into close proximity. The electric charge of droplets coated by LF‐SPI (≈+10 mV pH 5) was lower than that of primary emulsion (pH 7), thereby the multilayer emulsion was more sensitive to salt concentration. Overall, the two emulsions all exhibited weak stability to high NaCl concentrations, exhibiting great change in droplet size from 100 to 500 mM NaCl.

### 3.4 Influence of protein coatings on thermal stability

**FIGURE 4:**
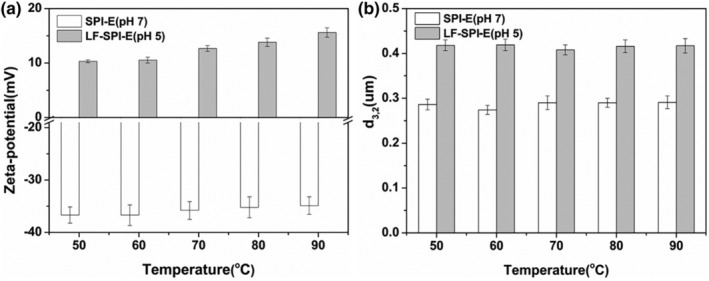
Effect of heating on the zeta‐potential (a) and droplet size (b) of emulsion (30 min). SPI, soy protein isolate.

Thermal treatment is one of the most common techniques used in food processing to assure product quality, avoid spoilage, and prevent food‐borne illnesses (Schmelz et al., 2011). To assess the thermal stability, the emulsions were held at temperatures ranging from 50 to 90°C for 30 min, and then cooled to ambient temperature before being analyzed (Figure 4). The effect of thermal treatment on the stability of primary and multilayer emulsions was determined at pH 7 and pH 5, respectively.

The zeta‐potential of primary emulsions was stable after the emulsion was heated at different temperatures (Figure 4a). The zeta‐potential of multilayer emulsions became slightly more positively charged with increasing temperature. Previous studies also found heated LF has higher surface charge than unheated protein, and the phenomenon may be caused by protein unfolding that leads to exposure of charged groups originally located in the hydrophobic interior (Mao et al., 2013; Schmelz et al., 2011). Primary emulsions remained relatively stable to droplet aggregation during heating when held at temperatures ranging from 50 to 90°C. This agrees with previous research that SPI‐coated lipid droplets were stable to aggregation when they were heated above their thermal denaturation temperature at low ionic strengths (Demetriades et al., 1997b).

### 3.5 Influence of protein coatings on chemical stability

**FIGURE 5:**
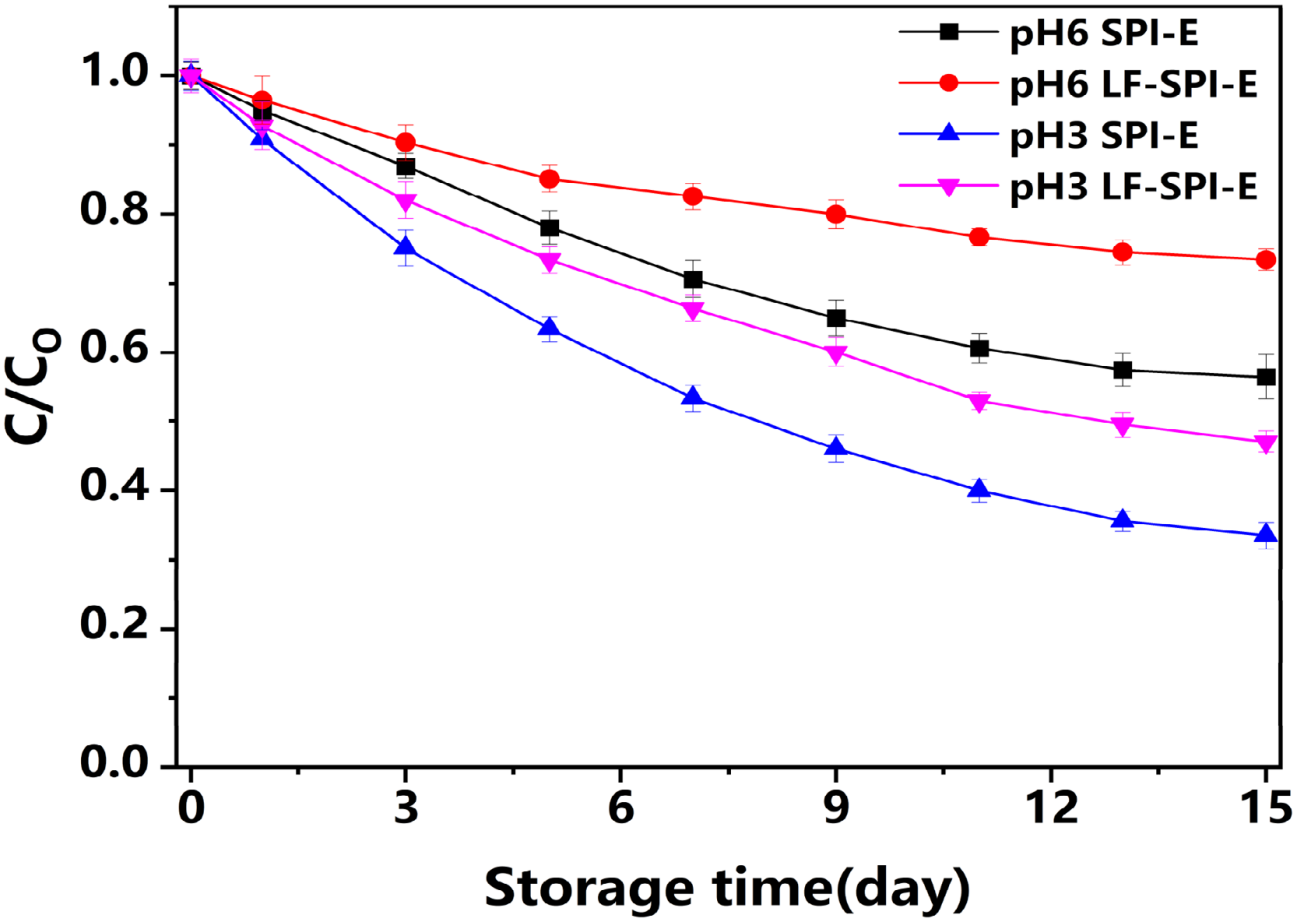
Effect of lactoferrin coating on degradation of carotenoids in emulsions. SPI, soy protein isolate.

Carotene is a polyunsaturated molecule that is highly susceptible to chemical degradation. It can be seen from the figure that after 15 days of accelerated oxidation at 37°C, the retention rate of carotenoids in multilayer emulsions was 73.4 ± 1.5% at pH 6 and 47.1 ± 1.6% at pH 3, both of which were much higher than the retention rate of carotenoids in the primary emulsions (Figure 5). There was a rapid change in the total color difference in the primary emulsions at pH 3. These results suggested that the presence of LF within the emulsion‐based delivery systems was able to retard the chemical degradation of encapsulated carotenoids. This could be due to the ability of lactoferrin molecules to strongly bind iron ions (Fe^2+^ or Fe^3+^) since transition metals are known to catalyze oxidation reactions. Previous studies have also shown that lactoferrin can inhibit the production of hydroperoxides and hexanaldehyde in corn oil emulsion, and the antioxidant activity gradually increases with the increase in lactoferrin concentration (Huang et al., 1999). Studies have also found that the presence of LF in the interfacial layer of milk droplets will hinder the chemical degradation of carotene in milk droplets (Mao et al., 2013). This may be due to the LF molecule's ability to strongly chelate metal ions, while transition metal ions catalyze oxidation reactions (Boon et al., 2009; Waraho et al., 2011). The multilayer emulsion system may have a thicker interfacial layer at a certain pH, or it may have a repulsive effect on metal ions. Compared with the primary emulsion, the degradation of carotenoids in the multilayer emulsion is more strongly inhibited.

### 3.6 Influence of protein coatings on oil digestion and bioaccessibility of carotenoids

**FIGURE 6:**
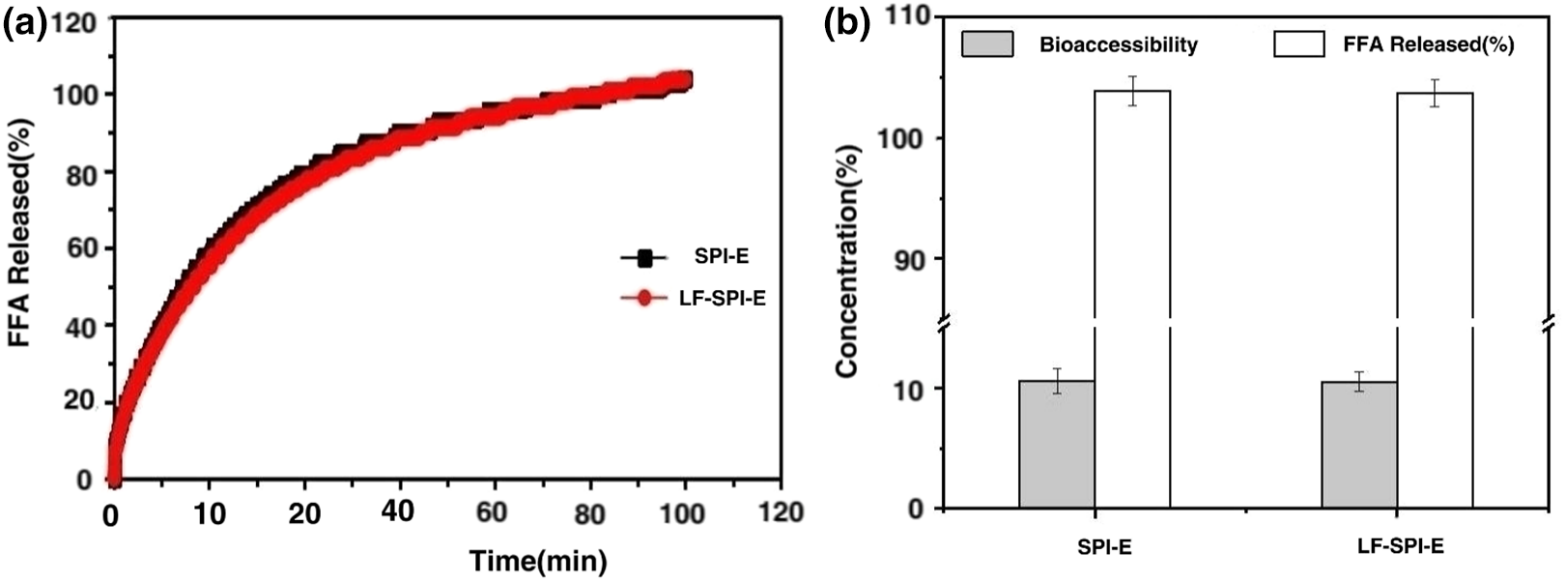
Effect of lactoferrin coating on FFAs released (a) and bioaccessibility of carotenoids (b). FFAs, free fatty acids; SPI, soy protein isolate.

The amount of free fatty acids released during oil digestion was determined by pH‐stat titration to study the effect of protein layer on oil digestion. FFAs release gradually increases with the time of digestion. After 2 h in vitro–simulated digestion, the FFAs release of primary emulsion and LF‐SPI emulsion was 103.9% and 103.7%, respectively (Figure 6a). There was no significant difference in the degree of oil digestion. The FFAs release rate of primary emulsion and LF‐SPI emulsion is basically similar, mainly because the interfacial layer of these two kinds of droplets is composed of proteins. Studies have pointed out that the particle size of SPI‐stable emulsions increases significantly after digestion in simulated gastric juice (Malaki Nik et al., 2011). This is mainly due to the reaction between pepsin and the protein layer at the interface of droplets, which exposes the oil droplets and causes them to aggregate and merge to form larger particles. The larger the oil droplet, the smaller the surface area, which is not conducive to the trypsin reaction, so the rate of oil hydrolysis is slow (Yi et al., 2014).

After simulated gastrointestinal digestion, the carotenoid content in the digestive and micellar phases was determined. Previous studies have shown that ingredients in simulated gastric juices will react with substances on the surface of oil droplets (Bauer et al., 2005). The bioaccessibility of carotenoids in the SPI emulsion and multilayer is 10.6 ± 1.03% and 10.5 ± 0.8% (Figure 6b). This showed that LF layer does not affect bioaccessibility because lactoferrin as a protein will reacts quickly with proteases in simulated gastric juices. The low bioaccessibility of the emulsion may be due to the presence of pepsin enzymolysis of the adsorbed protein layer, and then the oil droplets merge and aggregate and have a larger particle size when entering the small intestine for digestion. It has been reported that the bioavailability of carotene increases with the decrease in particle size (Salvia‐Trujillo et al., 2013), so the particle size of the emulsion when it enters the small intestine for digestion will affect the final bioaccessibility.

## 4 CONCLUSIONS

In order to improve the physical chemistry stability of SPI primary emulsion, a lactoferrin coating was formed on the surface of SPI emulsion droplets by interfacial electrostatic deposition. The stable multilayer emulsion can be formed when the content of lactoferrin is 0.5% at pH 5. The results showed that the LF protein greatly improved the environmental stability of the primary emulsion and modified the interfacial charge characteristics of the emulsion droplets. The lactoferrin layer did not hinder the digestion of oil and the bioaccessibility of carotenoids. In a word, LF‐SPI emulsion has better physical stability than the primary emulsion. Therefore, the selection of suitable emulsifier has a great influence on the properties of the emulsion. The rational design of the structure and composition of lipid droplet interfaces offer the possibility to modify emulsion physical and chemical stability.

## AUTHOR CONTRIBUTIONS

**Chunlan Zhang:** Data curation (equal); funding acquisition (equal); resources (equal); writing – original draft (equal); writing – review and editing (equal). **Mengyao Du:** Formal analysis (equal); writing – original draft (equal). **Bin Li:** Conceptualization (equal); investigation (equal); methodology (equal); supervision (equal).

## CONFLICT OF INTEREST STATEMENT

The authors declare that they do not have any conflicts of interest.

## Data Availability

The data that support the findings of this study are available on request from the corresponding author.
